# Unpaired Low-Dose CT Denoising Network Based on Cycle-Consistent Generative Adversarial Network with Prior Image Information

**DOI:** 10.1155/2019/8639825

**Published:** 2019-12-07

**Authors:** Chao Tang, Jie Li, Linyuan Wang, Ziheng Li, Lingyun Jiang, Ailong Cai, Wenkun Zhang, Ningning Liang, Lei Li, Bin Yan

**Affiliations:** PLA Strategy Support Force Information Engineering University, Zhengzhou, Henan Province 450001, China

## Abstract

The widespread application of X-ray computed tomography (CT) in clinical diagnosis has led to increasing public concern regarding excessive radiation dose administered to patients. However, reducing the radiation dose will inevitably cause server noise and affect radiologists' judgment and confidence. Hence, progressive low-dose CT (LDCT) image reconstruction methods must be developed to improve image quality. Over the past two years, deep learning-based approaches have shown impressive performance in noise reduction for LDCT images. Most existing deep learning-based approaches usually require the paired training dataset which the LDCT images correspond to the normal-dose CT (NDCT) images one-to-one, but the acquisition of well-paired datasets requires multiple scans, resulting the increase of radiation dose. Therefore, well-paired datasets are not readily available. To resolve this problem, this paper proposes an unpaired LDCT image denoising network based on cycle generative adversarial networks (CycleGAN) with prior image information which does not require a one-to-one training dataset. In this method, cyclic loss, an important trick in unpaired image-to-image translation, promises to map the distribution from LDCT to NDCT by using unpaired training data. Furthermore, to guarantee the accurate correspondence of the image content between the output and NDCT, the prior information obtained from the result preprocessed using the LDCT image is integrated into the network to supervise the generation of content. Given the map of distribution through the cyclic loss and the supervision of content through the prior image loss, our proposed method can not only reduce the image noise but also retain critical information. Real-data experiments were carried out to test the performance of the proposed method. The peak signal-to-noise ratio (PSNR) improves by more than 3 dB, and the structural similarity (SSIM) increases when compared with the original CycleGAN without prior information. The real LDCT data experiment demonstrates the superiority of the proposed method according to both visual inspection and quantitative evaluation.

## 1. Introduction

X-ray computed tomography (CT) is one of the most significant imaging modalities in modern hospitals and clinics. However, the risk of radiation in CT induces genetic, cancerous, and other diseases and has become a critical concern to patients and operators [1–3]]. A common and effective strategy to alleviate the risk is to achieve low-dose CT (LDCT) imaging by reducing the tube current during scanning and consequently decreasing the number of photons received by the detector. The dose reduction increases noise and artifacts in reconstructed CT images, thereby severely degrading the image quality and jeopardizing the clinical diagnosis. To solve this problem, researchers have proposed various noise-reduction strategies, including iterative reconstruction (IR) [4, 5], sinogram domain denoising [6–9], and image domain postprocessing [10–12].

Over the past decades, researchers have focused on developing new iterative algorithms for LDCT image reconstruction. In general, these algorithms optimize an objective function, which incorporates a system model [13, 14], a statistical noise model, and prior information in the image domain [4, 15, 16]. Well-known image priors consist of total variation (TV) and its variants [17–19], dictionary learning [20, 21], and wavelet frame [22]. These iterative reconstruction algorithms exhibit satisfactory performance in improving image quality, but their computational burden and sensitive parameters limit their practical applications.

Image postprocessing is more computationally efficient compared with iterative reconstruction, which has spawned a lot of simple and effective methods. Nonlocal means (NLM) filtering methods estimate noise components by using multiple patches extracted at different locations in the image [23] and have been widely used for CT [24]. Motivated by compressed sensing methods, an adaptive K-SVD method [25] was proposed to reduce artifacts in CT reconstructions. The block matching 3D (BM3D) method is also an outstanding method for image postprocessing in CT imaging fields [26, 27]; this method exploits similarities in image blocks. These traditional postprocessing methods have improved the quality of CT images; however, the results often undergo edge blurring and/or residual artifacts given the nonuniform distribution of reconstruction noise.

More recently, several supervised machine learning approaches have been proposed for noise reduction in LDCT. These methods usually reveal a relation between the pixel value in the LDCT image and the pixel value at the same location in a corresponding NDCT image by training with paired images. Chen et al. [28] designed a deep convolutional neural network (CNN) to map LDCT images toward its relative normal-dose counterparts in a patch-by-patch manner. Kang et al. [29] used a similar method but adopted CNN to directional wavelet transform of CT images. Then, more complex networks were proposed to handle the LDCT denoising problem such as the residual encoder-decoder convolutional neural network (RED-CNN) in [30], which achieves competitive performance relative to state-of-the-art methods in clinical cases.

Although the abovementioned networks presented impressive denoising results, they all belong to the end-to-end network, which typically utilizes mean squared errors (MSE) between the network output and the ground truth as loss function. However, recent studies [31, 32] indicated that this per-pixel MSE often suffers from oversmoothed edges and loss of details. MSE-based approaches tend to take the mean of high-resolution patches by using Euclidean distance rather than geodesic distance. Given that the medical images usually lie in a highly nonlinear manifold [33], the algorithm is prone to neglect subtle details that are vital for clinical diagnosis when it tries to minimize per-pixel MSE. To overcome the limitations of per-pixel regression in noise reduction, the generative adversarial network (GAN) [34] based on adversarial loss is introduced to medical image reconstruction. In 2017, Wolterink et al. [35] were the first to apply the GAN for cardiac CT image reconstruction. And, Yang et al. [36] utilized a GAN with Wasserstein distance (WGAN). In order to enhance the capability of noise reduction, perceptual loss is simultaneously used to optimize the loss function. Yi and Babyn [37] combined an adversarial trained network and a sharpness detection network to mitigate noise in LDCT and achieved satisfactory performance. Hence, a general framework for estimating generative models by using an adversarial process has shown outstanding performance in medical image reconstruction.

The above-mentioned denoising networks usually require spatially paired counterparts. However, in medical imaging, well-paired counterparts are difficult to obtain. For example, in LDCT imaging, continuously scanning a patient twice in normal and low dose is impossible under normal circumstances. The shortage of paired data has been one of the factors that restrict the wide application of deep learning in low-dose CT reconstruction. Recently, unsupervised variants of GANs, such as CycleGAN [38] and DualGAN [39], have been proposed for mapping different domains without matching data pairs. Motivated by their success in image processing, unpaired GANs have been successfully applied to CS-MRI reconstruction [40] and CT synthesis based on MR images [41, 42]. For LDCT reconstruction, well-paired clinical scans acquired at different dose levels are not readily available. Even if we obtain the same patient data at different dose levels, the data are difficult to match perfectly due to physical activity and the inevitable slight movement of the scanning position, which may affect the denoising ability of the networks.

**Figure 1:**
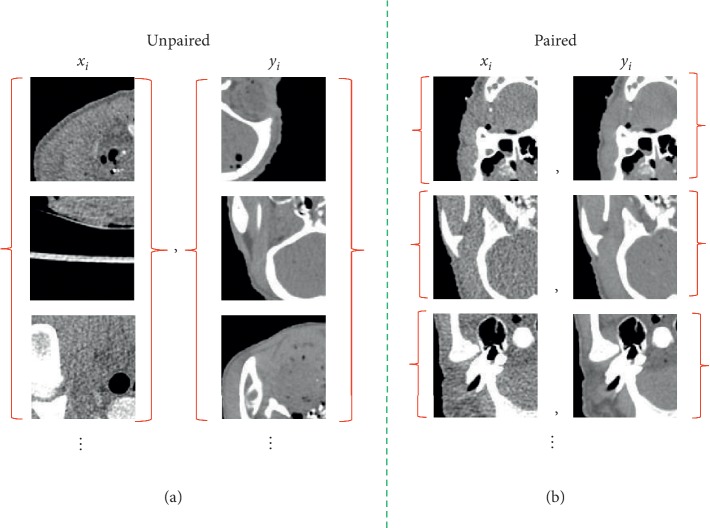
Schematic diagram of (a) unpaired dataset and (b) paired dataset.

In this study, we propose an unpaired LDCT denoising network based on CycleGAN with prior image information. In the proposed network, the design of cycle-consistent structure impels the network to learn the mapping relationship between the LDCT image collection and NDCT image collection (Figure 1(a)), rather than an image pair of an LDCT image and NDCT image (Figure 1(b)). Therefore, the proposed network does not need a one-to-one corresponding training dataset and can learn with the unpaired dataset. Meanwhile, the prior image information extracted from the preprocessed image by using LDCT is introduced into the network to supervise the generation of content and ensure correspondence of the image content. The map of image collections through cyclic loss and the supervision of content through prior image loss confer our proposed method to produce results that have not only lower noise but also accurate details.

## 2. Methods

### 2.1. Noise Reduction Model

In LDCT imaging, serious noise typically occurs in CT images as the number of photons received by the detector decreases. One of the effective ways to improve the image quality is designing a tailored network to make the input LDCT images as close as possible to the NDCT images. This process can be classified as an image denoising problem, which can be described by the following model:

(1) $$\begin{array}{c} G:x⟶y, \end{array}$$

where *x* ∈ *R*^*N*×*N*^ denotes an LDCT image and *y* ∈ *R*^*N*×*N*^ denotes the corresponding NDCT image. The goal of the noise reduction process is to obtain a transformation *G* that maps *x* to *y*.

In this process, *x* can be seen as a sample from the LDCT distribution *P*_*l*_ and *y* can be seen as a sample from the NDCT distribution *P*_*n*_. The denoising process transforms *x* to a certain distribution *P*_*g*_. And, the denoising process aims to determine an optimal *G* to make *P*_*g*_ close to *P*_*n*_. However, in the reconstructed LDCT images, noise is complicated and uniformly distributed over the whole image; as such, distributions *P*_*l*_ and *P*_*n*_ have no explicit mathematical relationship up to date [36]. The traditional methods usually have difficulty in denoising LDCT images. For deep learning-based methods, the uncertainty of a noise model can be ignored due to the learning capability of high-level features and presentation of data distribution by the CNN. Therefore, designing a tailored CNN is an effective method to suppress noise in LDCT and improve image quality.

### 2.2. Introduction of CycleGAN

In 2017, Zhu et al. [38] proposed an unpaired network named CycleGAN, which has gained extensive attention. This network can capture the special characteristics of one image collection and figure out how these characteristics could be translated into other image collection without using any paired training examples; this network has been successfully utilized in style transfer, object transfiguration, season transfer, and photo enhancement.

**Figure 2:**
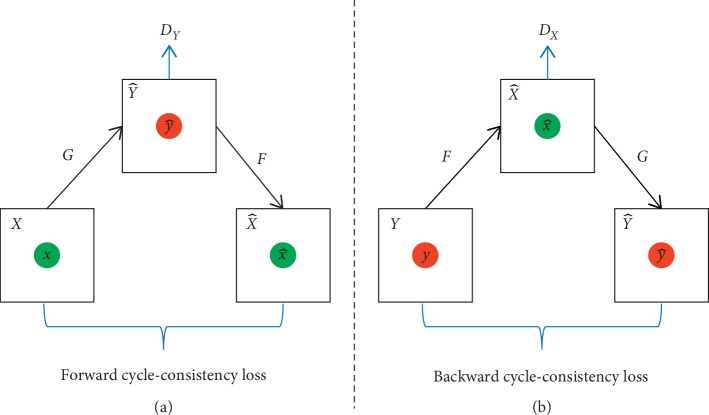
Structure diagram of CycleGAN. (a) Forward cycle-consistency loss. (b) Backward cycle-consistency loss.

Under the assumption that there is some underlying relationship between the source domain *X* and target domain *Y*, the goal of CycleGAN is to learn mapping *G* : *X*⟶*Y* so that the distribution of image from *G*(*x*) is indistinguishable from the distribution of image from domain *Y*. This network includes two mapping functions, namely, *G* : *X*⟶*Y* and *F* : *Y*⟶*X* and also introduces two discriminators, namely, *D*_*X*_ and *D*_*Y*_. Discriminator *D*_*Y*_ aims to distinguish between translated samples *G*(*x*) and real samples *y*. Discriminator *D*_*X*_ aims to distinguish between translated samples *F*(*y*) and real samples *x*. In theory, adversarial training can identify mappings *G* and *F* that produce outputs identically distributed as target domains *Y* and *X* [34]. However, with its large sufficient capacity, a network can map the same set of input images to any random permutation of images in the target domain, where any of the learned mappings can induce an output distribution that matches the target distribution. Therefore, adversarial loss alone cannot guarantee that the learned function can map individual input *x* to desired *y*. To further reduce the space of possible mapping functions, the mapping functions *G* and *F* should be cycle consistent. As shown in Figure 2(a), for each image *x* from domain *X*, the image translation cycle should be able to bring *x* back to the original image: *x*⟶*G*(*x*)⟶*F*(*G*(*x*)) ≈ *x*, which is named as forward cycle consistency. Similarly, as demonstrated in Figure 2(b), for each image *y* from domain *Y*, the image translation cycle should be able to bring *y* back to the original image: *y*⟶*F*(*y*)⟶*G*(*F*(*y*)) ≈ *y*, which is named as backward cycle consistency. The abovementioned behavior can be incentivized by cycle-consistency loss.

(2) $$\begin{array}{c} L_{\text{cyc}}\left(G,F\right)=E_{x∼P_{\text{data}}\left(x\right)}\left[{\left\|F\left(G\left(x\right)\right)-x\right\|}_{1}\right]+E_{y∼P_{\text{data}}\left(y\right)}\left[{\left\|G\left(F\left(y\right)\right)-y\right\|}_{1}\right], \end{array}$$

where *P*_data(*x*)_ is the distribution of *x* and *P*_data_(*y*) is the distribution of *y*_._

In LDCT imaging, although the projection data contain a lot of noise, it is usually complete. Therefore, the reconstructed images still contain useful information that is basically consistent with corresponding NDCT images. This indicates that there is a close relationship between the LDCT image and NDCT image and satisfies the basic assumption of CycleGAN. Thus, this study considers using this unpaired network for LDCT image reconstruction.

Based on its structure, CycleGAN mainly focuses on the map of distributions. This network is better at the overall conversion of images and may overlook the correspondence of details. However, for LDCT noise reduction, the outputs should not only appear similar to an NDCT image but also retain details as much as possible; more importantly, the output must not contain false information, which may cause misdiagnosis. These issues require additional supervision and restraint to the network in the training process to make it more suitable for LDCT reconstruction. In this study, we consider incorporating prior image information into the network to guarantee content correspondence and prevent the generation of fake details during denoising process.

### 2.3. Unpaired Denoising Network Based on CycleGAN with Prior Information

**Figure 3:**
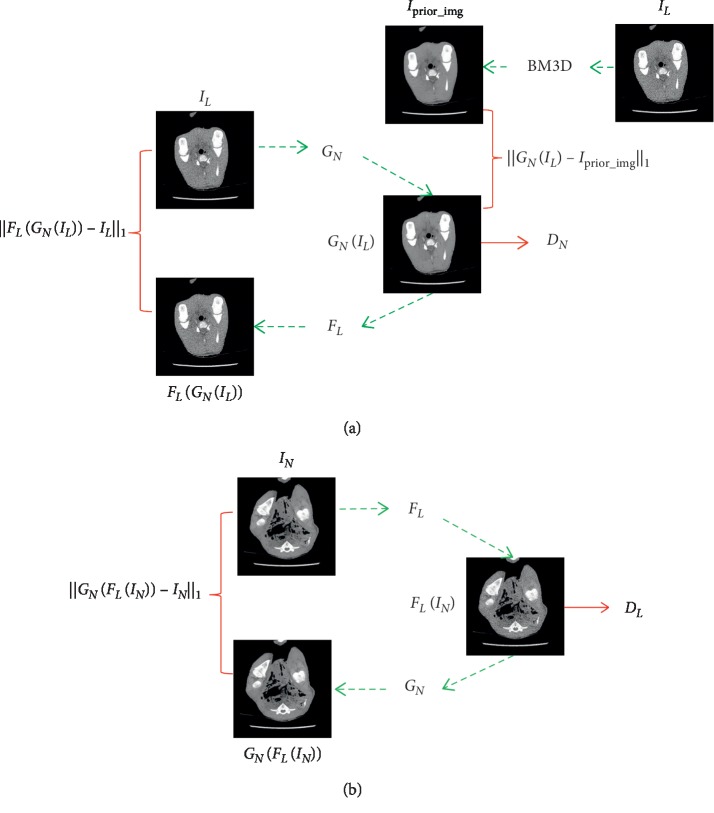
Overview of the proposed method. (a) Forward cycle. (b) Backward cycle.

**Figure 4:**
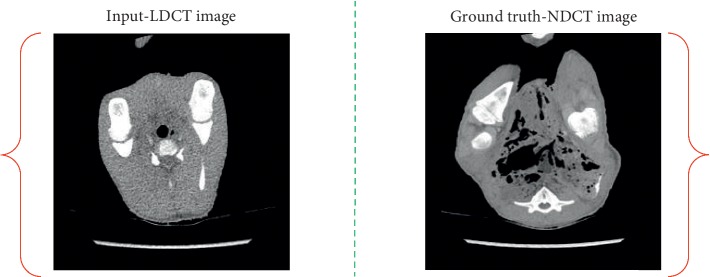
One input-ground truth pair of the proposed unpaired network.

Figure 3 shows an overview of our proposed method. The network contains forward and backward cycles and two generators and discriminators. In order to more clearly illustrate the training mechanism of the proposed network, we randomly selected an LDCT image from LDCT image collection and an NDCT image from the NDCT image collection as the input-ground truth pair. Note that the LDCT image is not corresponding to the NDCT image, as shown in Figure 4.

In the forward cycle, generator *G*_*N*_ is trained to generate images that are as close to corresponding NDCT images as possible (Figure 3(a)). Generator *F*_*L*_ is trained to translate the resulting image *G*_*N*_(*x*) back to the corresponding LDCT image. In the backward cycle, generator *F*_*L*_ is trained to generate images that are as close to corresponding LDCT images as possible (Figure 3(b)). Generator *G*_*N*_ is trained to translate the resulting image *F*_*L*_(*y*) back to the NDCT image. In the training process of network, the discriminators *D*_*N*_ and *D*_*L*_ are used to estimate the probability that the sample is from the real image rather than generating image. At the same time, the generators *G*_*N*_ and *F*_*L*_ attempt to generate images that are not easily distinguishable by the discriminators. This paper utilizes the adversarial loss [43] as the objective function to train the “game” process:

(3) $$\begin{array}{c} L_{{\text{GAN}}_{N}}\left(G_{N},D_{N},X,Y\right)=E_{y∼p_{\text{data}}\left(y\right)}\left[\log\;D_{N}\left(y\right)\right]+E_{x∼p_{\text{data}}\left(x\right)}\left[\log\left(1-D_{N}\left(G_{N}\left(x\right)\right)\right)\right],\\ L_{{\text{GAN}}_{L}}\left(F_{L},D_{L},X,Y\right)=E_{x∼p_{\text{data}}\left(x\right)}\left[\log\;D_{L}\left(x\right)\right]+E_{y∼p_{\text{data}}\left(y\right)}\left[\log\left(1-D_{L}\left(F_{L}\left(y\right)\right)\right)\right]. \end{array}$$

In order to reduce the feasible domain space of the mapping functions, the cycle consistency is introduced to further constrain the training process of the network so that the network can be trained under unpaired data:

(4) $$\begin{array}{c} L_{\text{cyc}}\left(G_{N},F_{L}\right)=E_{x∼P_{\text{data}}\left(x\right)}\left[{\left\|F_{L}\left(G_{N}\left(x\right)\right)-x\right\|}_{1}\right]+E_{y∼P_{\text{data}}\left(y\right)}\left[{\left\|G_{N}\left(F_{L}\left(y\right)\right)-y\right\|}_{1}\right]. \end{array}$$

For this cycle consistency network, the performance of a generator requires the indirect supervision through the results of another generator. For example, in the forward cycle, in addition to discriminator *D*_*N*_, the performance of generator *G*_*N*_ also needs to be supervised by the results *F*_*L*_(*G*_*N*_(*x*)) of another generator *F*_*L*_. This mechanism does not guarantee the accuracy of the final input and may lead to the occurrence of fake details. For LDCT image reconstruction, the accuracy of the results is critical, and if false information is generated, it may cause misdiagnosis, leading to serious consequences. These circumstances require direct constraints to the generators, especially to generator *G*_*N*_, which directly produces the desired outcome. Therefore, this paper incorporates the prior image information into the network to directly supervise the generator *G*_*N*_, as shown in Figure 3(a). For fear of changing the unpaired property of the network, the resulting images processed by BM3D method utilizing LDCT images are regarded as prior images. And, the mean absolute error (MAE) between the prior image and the generated image is introduced into the loss function to constrain the training of the network. The adversarial loss of the forward cycle can be written as

(5) $$\begin{array}{c} L_{{\text{GAN}}_{N}}^{p}\left(G_{N},D_{N},X,Y\right)=E_{y∼p_{\text{data}}\left(y\right)}\left[\log\;D_{N}\left(y\right)\right]+E_{x∼p_{\text{data}}\left(x\right)}\left[\log\left(1-D_{N}\left(x\right)\right)+\alpha {\left\|G_{N}\left(x\right)-I_{\text{prior\_img}}\right\|}_{1}\right], \end{array}$$

where *α* is the weight of the MAE. In the training process, the generator *G*_*N*_ tries to minimize the objective function (5) while the discriminator *D*_*N*_ tries to maximize it, that is, min_*G*_*N*__max_*D*_*N*__*L*_GAN_*N*__^*p*^(*G*_*N*_, *D*_*N*_, *X*, *Y*). We denote the proposed network as CycleGAN-BM3D.

Through the above analysis, the total loss function of our proposed method is

(6) $$\begin{array}{c} L\left(G_{N},F_{L},D_{N},D_{L}\right)=L_{{\text{GAN}}_{N}}^{p}\left(G_{N},D_{N},X,Y\right)+L_{{\text{GAN}}_{L}}\left(F_{L},D_{L},Y,X\right)+\lambda L_{\text{cyc}}\left(G,F\right), \end{array}$$

where *λ* is a nonnegative parameter used to balance the weight of the cycle consistency loss. The total objective function of the proposed CycleGAN-BM3D is

(7) $$\begin{array}{c} G_{N}^{*},F_{L}^{*}=\arg\underset{G_{N},F_{L}}{\min}\underset{D_{N},D_{L}}{\max}L\left(G_{N},F_{L},D_{N},D_{L}\right). \end{array}$$

### 2.4. Network Architecture

**Figure 5:**
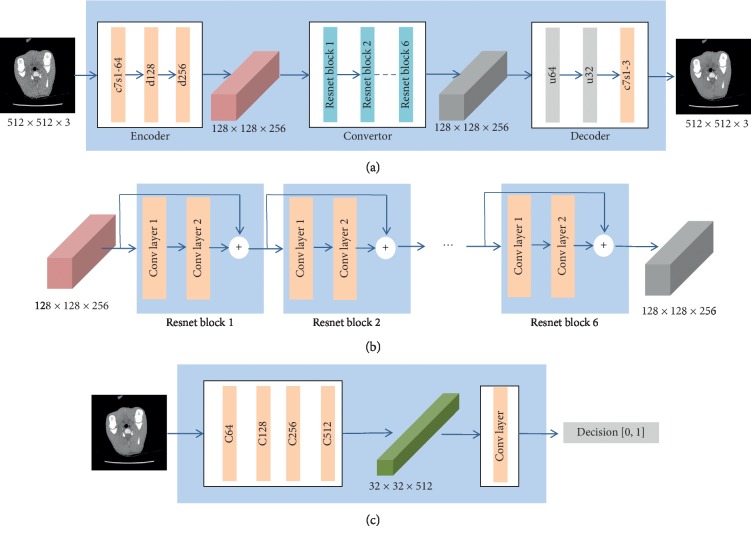
Network structure. (a) Diagram of generator's network structure. (b) Diagram of convertor's network structure. (c) Diagram of discriminator's network structure.

In the proposed method, the network of the generator, as shown in Figure 5(a), includes three submodules: encoder, convertor, and decoder. The encoder extracts the features from the input image utilizing CNN. The network of the encoder includes one 7 × 7 Convolution-InstanceNorm-ReLU layer with 64 filters and stride 1 denoted as c7s1-64, two 3 × 3 Convolution-InstanceNorm-ReLU layers with *k* filters and stride 2 in which *k* equals to 128 and 256, respectively. We denote these two layers as d128 and d256. The convertor, as shown in Figure 5(b), is used to convert feature vectors extracted from the source domain *X* to the target domain *Y*. The convertor contains six residual network (Resnet) blocks [44], and each block contains two 3 × 3 convolutional layers with 256 filters on both layers. The decoder includes three layers. The first two layers are 3 × 3 fractional-strided-Convolution-InstanceNorm-ReLU layers with stride 1/2 and 64 and 32 filters, respectively. We denote the two layers as u64 and u32. The third layer is a 7 × 7 Convolution-InstanceNorm-ReLU with 3 filters and stride 1 denoted as c7s1-3. The network of the discriminator, as shown in Figure 5(c), consists of five convolution layers. The first four layers are 4 × 4 Convolution-InstanceNorm-LeakyReLU layers with stride 2 and 64, 128, 256, and 512 filters, respectively. We denote them as C64, C128, C256, and C512. We use leaky ReLUs with slope 0.2. In the last layer, a 4 × 4 convolution layer with 1 filter and stride 1 is utilized to produce a one-dimensional output.

## 3. Experiments and Results

### 3.1. Experimental Dataset and Performance Evaluations

The CT images of a deceased piglet were selected as the experimental dataset to verify the performance of the proposed network. The images were scanned by a 64-slice multidetector GE Healthcare scanner (Discovery CT750 HD) by using 100 kV and 0.625 mm slice thickness. Five different tube currents were set to yield CT images with different dose levels. The specific scanned parameters and effective dose of different tube currents are listed in Table 1.

**Figure 6:**
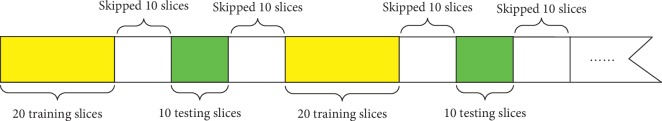
Schematic diagram of data preparation. The yellow rectangular blocks represent the training data, the green rectangular blocks represent the test data, and the white rectangular blocks represent the skipped slices.

In each dose level, 906 images with size 512 × 512 were acquired. As shown in Figure 6, we partitioned the slices by taking oneʼs data and then skipping 10 slices. We finally obtained 360 images reconstructed by FBP using the projection data of 5% dose as the noisy dataset, that is, source collection *X*. And, 180 images were obtained for testing. The normal dose dataset utilized consists of 360 images, which are corresponding to the noisy dataset and obtained from the NDCT images constructed by FBP. The normal dose dataset is the target collection *Y*. Given the unpaired property of the proposed network, the training images and the ground truth do not require a one-to-one correspondence. The number of two image datasets can also be different. In the training stage, we performed an unpaired operation for the inputs and labels of the network. In addition, each image was divided into sixteen 128 × 128 images to enlarge the training dataset to 5760 images.

For comparison, we selected some representative traditional methods and other networks.

#### 3.1.1. BM3D

This method exhibits outstanding performance in noise reduction over other traditional image denoising methods.

#### 3.1.2. Original CycleGAN

This network is trained without the constraint of prior image to test the supervised effect of the prior image. The input dataset and ground truth dataset are the same as the proposed method CyclGAN-BM3D, which are not one-to-one correspondence.

Structural similarity (SSIM) [45], peak signal-to-noise ratio (PSNR), and normalized mean absolute distance (NMAD) are selected as measures of reconstruction quality for the quantitative assessment of the proposed network and abovementioned contrast algorithms. Specifically, PSNR and NMAD are defined as follows:

(8) $$\begin{array}{c} \text{PSNR}=10\;\log_{10}\left(\frac{{\text{MAX}}^{2}\left(f\right)}{1/N\sum_{i=1}^{N}{\left|f\left(i\right)-f_{0}\left(i\right)\right|}^{2}}\right),\\ \text{NMAD}=\frac{\sum_{i=1}^{N}\left|f\left(i\right)-f_{0}\left(i\right)\right|}{\sum_{i=1}^{N}\left|f\left(i\right)\right|}, \end{array}$$

where *f* and *f*_0_ represent the denoising image and ideal image, respectively, *i* is the pixel in the image, and *N* is the total number of pixels in the image. A higher PSNR indicates that the image is of higher quality. The NMAD value close to 0 indicates small differences between the ideal image and the reconstructed results. In general, SSIM ≤ 1 and SSIM = 1 indicate the exact theoretical reconstruction.

### 3.2. Implementation Details

In the training process, the negative log likelihood objective of the first two items in *L*_GAN_*N*__ and *L*_GAN_*L*__ is replaced by least squares loss to stabilize the proposed model [46]. After the replacement, we train *G*_*N*_ to minimize

(9) $$\begin{array}{c} E_{x∼p_{\text{data}}\left(x\right)}\left[{\left(D_{N}\left(G_{N}\left(x\right)\right)-1\right)}^{2}+\alpha {\left\|G_{N}\left(x\right)-I_{\text{prior\_img}}\right\|}_{1}\right]. \end{array}$$

We also train *F*_*L*_ to minimize

(10) $$\begin{array}{c} \min{\;E}_{y∼p_{\text{data}}\left(y\right)}\left[{\left(D_{L}\left(F_{L}\left(y\right)\right)-1\right)}^{2}\right]. \end{array}$$

For discriminators *D*_*N*_ and *D*_*L*_, the objectives are

(11) $$\begin{array}{c} \min\;E_{y∼p_{\text{data}}\left(y\right)}\left[{\left(D_{N}\left(y\right)-1\right)}^{2}\right]+E_{x∼p_{\text{data}}\left(x\right)}\left[D_{N}\left(G{\left(x\right)}^{2}\right)\right],\\ \min{\;E}_{x∼p_{\text{data}}\left(x\right)}\left[{\left(D_{L}\left(x\right)-1\right)}^{2}\right]+E_{y∼p_{\text{data}}\left(y\right)}\left[D_{L}\left(F_{L}{\left(y\right)}^{2}\right)\right]. \end{array}$$

**Figure 7:**
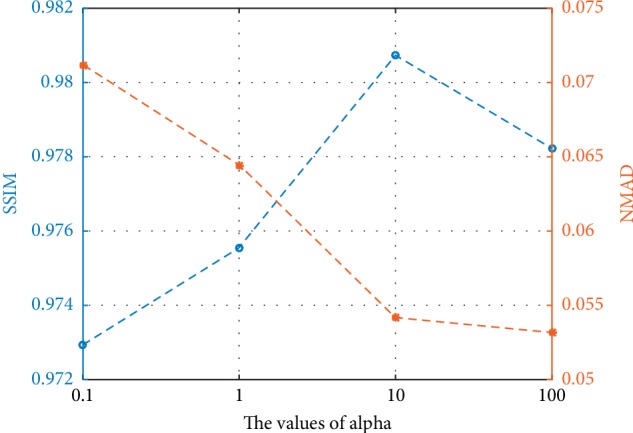
Average SSIM and NMAD of 10 images in different values of *α*. The blue line indicates the SSIM curve and the orange line represents the NMAD curve.

For the setting of parameters, *λ* is set as 10. Adam solver with a batch size of 1 is selected as the optimizer to optimize the networks. We keep the same learning rate for the first 10,000 epochs and linearly decay the rate to zero over the next 10,000 epochs. The proposed network in this paper trained 40,000 epochs. *α* is an important parameter for controlling the weight of prior information during training. When the value of *α* is too small, the effect of prior information will be negligible and cause minimal improvement in image quality. By contrast, a very large *α* will overemphasize the role of prior information and, to some extent, limit the learning ability of the network itself. In this paper, a series of networks was trained by setting different values of *α* to determine a suitable value. For the sake of fairness, each network has the same parameters' setting, except *α*. We randomly selected 10 LDCT images to test the performance of different networks. The effect of *α* was quantitatively determined by plotting the average SSIM and NMAD of the denoising images in Figure 7.

From the curves of Figure 7, when *α*=10, the SSIM reaches the maximum, indicating that the denoising images are most similar to the NDCT images in structure, that is, under this circumstance, the network has the greatest ability to retain the details of the LDCT images. NMAD reflects the accuracy of denoising to some extent. Smaller NMAD indicates that the noise in the LDCT images is removed more completely. Considering the detail retention and noise reduction of the network, this paper set *α* as 10.

**Figure 8:**
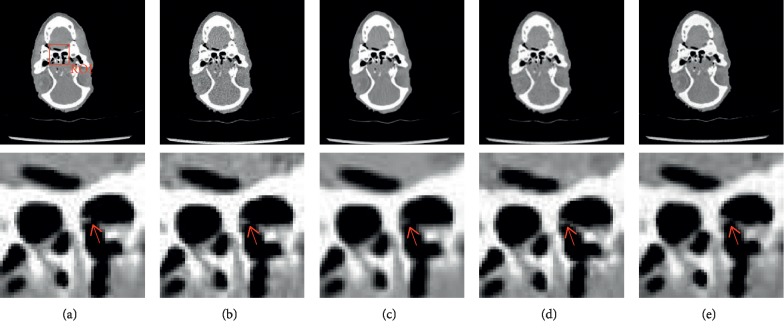
Results of (a) NDCT, (b) LDCT, (c) BM3D, (d) CycleGAN, and (e) CycleGAN-BM3D, respectively. The display widow is [800, 1300].

**Figure 9:**
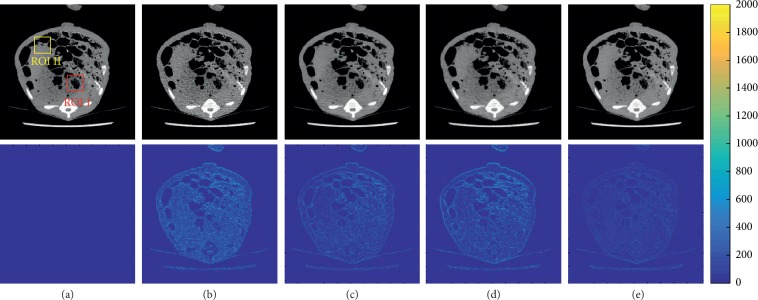
Results of (a) NDCT, (b) LDCT, (c) BM3D, (d) CycleGAN, and (e) CycleGAN-BM3D, respectively. The display widow is [800, 1300].

**Figure 10:**
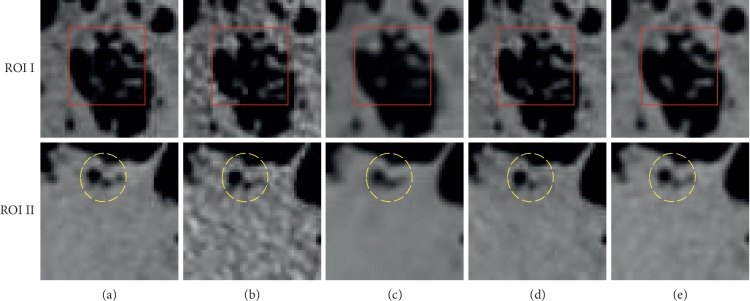
Zoomed-in ROIs of slice 2. The first column is the NDCT (a). The following columns are the results from (b) LDCT, (c) BM3D, (d) CycleGAN, and (e) CycleGAN-BM3D, respectively. The display widow is [800, 1300].

To analyze the potential denoising capability of selected algorithms and networks, two representative slices and the corresponding zoomed regions of interest (ROIs) are shown in Figures 8–10, respectively.

As shown in Figure 8, when using traditional methods (BM3D) or deep learning-based methods, the noise that appears in the LDCT image is suppressed to varying degrees. The classic BM3D method has an outstanding noise suppression effect but makes the processed image oversmoothed so that some vital details disappear. As indicated by the red arrows in ROI, the results of BM3D lose some information of the bone. Although the result of the original CycleGAN is not oversmoothed, it shows fake details, connecting the unconnected bones in the NDCT image. The CycleGAN-BM3D, which introduces prior image information, does not have redundant details and retains the information that should be retained.

Figure 9 shows the overall second slice of different methods and the corresponding absolute difference images between NDCT images and the resulting images. In the difference images, the darker the color is, the smaller the error will be. It can be clearly observed that the result of CycleGAN-BM3D has the smallest difference.

For further analysis of image details, two regions were selected as ROIs, which are shown in Figure 10. In ROI I, the tissue pointed by red squares is smeared out in the BM3D images but is easily identifiable in the CycleGAN and CylceGAN-BM3D images. As marked by the yellow ellipses in ROI II, the three black holes in the results of BM3D and CycleGAN are blurred and inseparable but are recognizable in the result of CycleGAN-BM3D. Furthermore, the smooth area below the ROI II of CycleGAN-BM3D is the most similar to the NDCT image.

Based on the visual effect, the proposed network CycleGAN-BM3D can not only better suppress noise but also retain more details than the other networks. More importantly, after adding the constraint of prior information, CycleGAN-BM3D can effectively prevent the generation of fake details compared with the original CycleGAN without prior information.

For quantitative analysis, the average of PSNR, SSIM, and NMAD was calculated for 180 slices in the test dataset to measure performance of the proposed method and the other compared methods. (Table 2).

In each evaluation item, the results with the best performance are marked black. CycleGAN-BM3D ranks first in terms of SSIM even with the unpaired training dataset. As such, the results of CycleGAN-BM3D are the most structurally similar to the NDCT images. In terms of PSNR and NMAD, CycleGAN-BM3D also exhibits satisfactory performance, indicating that the noise removal is relatively clean. Compared with the other algorithms, CycleGAN shows the worst performance because it mainly focuses on mapping the data distribution from the LDCT to NDCT, and cyclic loss may not be enough to supervise the generation of details and suppression of noise. This method needs to add supervision during training to improve image quality. The introduction of prior information in CycleGAN-BM3D enhances the constraints to the image content. That is, in the proposed CycleGAN-BM3D method, cyclic loss plays a role in distribution mapping and prior information loss is used to guarantee the relevance of the content. Therefore, the proposed method demonstrates good performance in noise suppression and detail preservation. We note that the numerical results of BM3D are in the front rank. From the visual effect, the noise removal of BM3D is complete and the main information of the image is basically retained, as much, BM3D has a high quantitative evaluation result. That finding is the reason why we choose the result of BM3D as the prior information.

## 4. Discussion and Conclusion

In the modern CT imaging field, the hidden risk of radiation dose has increased the demand for LDCT. However, LDCT images often suffer from serious noise, which degrades image quality and troubles clinical diagnosis. In the past two years, deep neural network provides a new idea for LDCT noise reduction. Most of the existing neural networks for LDCT reconstruction usually require well-matched datasets for network training. However, the well-matched CT images of different dose levels are difficult to obtain. This may affect the performance of networks and lead to blurred details or fake information in the resulting images.

To improve the quality of LDCT image and broaden the application of neural networks in LDCT noise reduction, this paper proposed an unpaired network based on CycleGAN with prior image information. In contrast to existing denoising networks, the proposed network can be trained by unpaired datasets, thereby alleviating the limitation of paired dataset requirement. Most GANs used to reduce noise mainly focus on the distribution mapping from LDCT to NDCT, and this process may overlook the accurate content correspondence. To enhance the constraint to the content and prevent producing fake details, we incorporate the prior image processed by BM3D into CycleGAN to supervise the generation of image content. In the experiment of real data, visual inspection demonstrated that the proposed method can suppress noise in the LDCT image and prevent the generation of fake details. The result of quantitative evaluations indicated that, after incorporating prior information, the PSNR improved more than 3 dB and SSIM also increased compared with the original CycleGAN without prior information. The results of qualitative and quantitative evaluations indicated that the proposed method exhibits reasonable performance and outperforms the original CycleGAN when applied to LDCT reconstruction.

The validity of prior information affects the performance of the proposed method. In this work, the LDCT images processed by traditional methods are obtained as prior information, which is a simple and efficient way. In the future, we intend to explore other representative shared features between LDCT and NDCT images as prior information to further improve the performance of the proposed network, such as the sparsity or sharpness information.

## Figures and Tables

**Table 1:** CT scanning protocol for the experiment.

	Normal dose	50% dose	25% dose	10% dose	5% dose
Tube current (mAs)	300	150	75	30	15
Effective dose (mSv)	14.14	7.07	3.54	1.41	0.71

**Table 2:** Quantitative evaluation of results by different algorithms on 180 slices in the test dataset.

	PSNR	SSIM	NMAD
LDCT	27.6514 ± 1.3862	0.8698 ± 0.0375	0.1124 ± 0.0174
BM3D	32.2185 ± 0.9599	0.9271 ± 0.0245	0.0681 ± 0.0087
CycleGAN	29.0833 ± 0.8661	0.9059 ± 0.0263	0.0890 ± 0.0096
CycleGAN-BM3D	**34.3577** **±** **0.1475**	**0.9798** **±** **0.0041**	**0.0583** **±** **0.0012**

## Data Availability

The data used in the study can be obtained from http://homepage.usask.ca/∼xiy525/publication/sagan/.
